# Prepubertal Screening for Testicular Adrenal Rest Tumors in Boys with Classic Congenital Adrenal Hyperplasia: Emerging Evidence and Practical Implications

**DOI:** 10.3390/children13081003

**Published:** 2026-07-29

**Authors:** Alice Ranieri, Vittorio Ferrari, Rita Ortolano, Egidio Candela, Luca Bernardini, Marcello Lanari, Federico Baronio

**Affiliations:** 1 Specialty School of Pediatrics, Alma Mater Studiorum, University of Bologna, 40126 Bologna, Italy; alice.ranieri3@studio.unibo.it; 2 Pediatric Unit, IRCCS Azienda Ospedaliero-Universitaria di Bologna, 40138 Bologna, Italy; rita.ortolano@aosp.bo.it (R.O.); egidio.candela@aosp.bo.it (E.C.); luca.bernardini16@unibo.it (L.B.); marcello.lanari@unibo.it (M.L.); federico.baronio@aosp.bo.it (F.B.); 3 Department of Medical and Surgical Sciences, Alma Mater Studiorum, University of Bologna, Via Massarenti 11, 40126 Bologna, Italy

**Keywords:** testicular adrenal rest tumors, congenital adrenal hyperplasia, adolescents, screening, testicular tumor, scrotal ultrasound, male infertility, 21-hydroxylase deficiency

## Abstract

Testicular adrenal rest tumors (TARTs) are a frequent and clinically relevant complication of congenital adrenal hyperplasia (CAH), particularly its classic forms due to 21-hydroxylase deficiency. Although histologically benign, these masses develop within the rete testis and can progressively compress the seminiferous tubules, leading to fibrosis and, when longstanding, permanent gonadal injury and obstructive azoospermia; they are the foremost cause of impaired fertility in men with classic CAH. Their frequency increases with age, from approximately 25% in childhood to 46% in adulthood. This narrative review summarizes current evidence on the pathogenesis, predisposing factors and diagnostic work-up of TARTs, focusing on two practical questions: the appropriate age to begin monitoring and the imaging tools best suited to it. Sustained ACTH excess is regarded as the dominant growth-promoting signal, while the salt-wasting phenotype, severe CYP21A2 variants and inadequate disease control emerge as the principal predisposing factors. Although current guidelines advise ultrasound monitoring from adolescence, a growing body of pediatric data shows that TARTs can be detected well before puberty in patients at greatest risk. Scrotal ultrasonography remains the first-line technique, complemented by CEUS, elastography and MRI in selected cases. We outline a pragmatic, risk-adapted monitoring scheme and argue that earlier, childhood assessment in the highest-risk patients may help to safeguard future fertility, an approach that still awaits prospective confirmation.

## 1. Introduction

Testicular adrenal rest tumors (TARTs) are a frequent complication in males with congenital adrenal hyperplasia (CAH), a group of autosomal recessive disorders caused by enzymatic defects in adrenal steroidogenesis, with 21-hydroxylase deficiency (21OHD) accounting for over 90% of cases [1]. Although benign, these lesions are clinically relevant: by progressively expanding within the rete testis, TARTs can compromise testicular function through seminiferous tubule obstruction and parenchymal compression; when longstanding, this process may culminate in fibrosis, irreversible testicular damage, obstructive azoospermia, and infertility, making TARTs the main cause of male subfertility in classic CAH [2,3]. CAH due to 21OHD is characterized by reduced cortisol production, chronic adrenocorticotropic hormone (ACTH) hypersecretion, accumulation of steroid precursors, and hyperandrogenism. The classic form is subdivided into salt-wasting (SW, <1% residual enzyme activity) and simple virilizing (SV, 1–2% residual activity); a non-classic form (NC, 20–50%) presents more mildly [4]. The global prevalence of TARTs in classic CAH ranges from 14% to 94%, depending on cohort age, detection methodology, and degree of hormonal control. Pooled data indicate a mean prevalence of around 37%, increasing from childhood and adolescence (25%) to adulthood (46%) [2]. This review synthesizes the available literature, addressing two key clinical questions: when to initiate screening and which diagnostic modalities to employ. The risk-stratified screening framework proposed in this review reflects a synthesis of the available observational evidence rather than formal clinical guidelines and should be interpreted as a complement to individualized clinical judgement pending prospective validation.

## 2. Literature Search Strategy

The literature was searched in PubMed/MEDLINE using combinations of the terms (“testicular adrenal rest tumor” OR “TART*”) AND (“congenital adrenal hyperplasia” OR “CAH”) AND (“male*” OR “men”), with a focus regarding screening and surveillance in patients with congenital adrenal hyperplasia; reviews, guideline-based recommendations and case reports were also considered. The search was restricted to English-language articles published in the last 15 years with a focus on the pediatric and adolescent male population; older studies were included when relevant to pathogenesis, histological staging, fertility outcomes, or genotype–phenotype correlations. Eligible publications included English-language original studies, reviews, clinical guidelines, and relevant case reports concerning male patients with CAH and reporting data on TART pathogenesis, prevalence, risk factors, diagnosis, imaging, surveillance, histology, or fertility outcomes. Publications without relevant data on male patients or TARTs and non-English-language articles were excluded. Titles, abstracts, and full texts were screened independently by two authors (A.R. and V.F.), with disagreements resolved by consensus. A total of 60 records were identified, of which 41 were included in the review.

## 3. Etiopathogenetic Characteristics of TARTs

### 3.1. Pathogenesis of TARTs

The classical hypothesis proposes that TARTs originate from ectopic adrenocortical cells that migrated with the testes during embryological development from the adrenogonadal primordium and are subsequently hyperstimulated by the chronically elevated ACTH characteristic of CAH [2]. This hypothesis is supported by the expression of adrenal steroidogenic markers and ACTH receptors in TART tissue, as well as by evidence that cultured TART cells respond to ACTH stimulation with steroid production in vitro [2,5]. An alternative, and possibly complementary, theory supported by molecular and transcriptomic data proposes that TARTs originate from still-unidentified pluripotent steroidogenic progenitor cells of the adrenogonadal primordium or urogenital ridge, capable of acquiring both adrenocortical and testicular features [2,6,7]. Accordingly, TART tissue has been shown to express adrenal markers, including CYP11B1, CYP11B2, and MC2R, together with testicular/Leydig-cell-related markers such as LHCGR, HSD17B3, and GATA4 [2,8]. The presence of receptors for ACTH, angiotensin II, and LH/hCG may partly explain the influence of multiple hormonal factors on tumor growth. ACTH remains the dominant trophic stimulus, supported by near-universal MC2R expression and the ACTH-responsiveness of cultured TART cells, whereas angiotensin II and LH/hCG receptors have also been reported in TART tissue, though their functional contribution to tumor growth remains less well established [2,5,8]. It has been proposed that early neonatal or childhood exposure to elevated ACTH may prevent the physiological regression of adrenal-like residual tissue, or of an as-yet unidentified ACTH-sensitive testicular progenitor-cell population, thereby expanding the pool of cells susceptible to later TART development during periods of inadequate hormonal control [9]. This hypothesis is consistent with the higher prevalence of TARTs in severe forms of CAH, in which ACTH excess may already be present in utero, and with the rarity of TARTs in acquired conditions characterized by ACTH elevation beginning only in adulthood, such as Addison’s disease or post-adrenalectomy states [2,7]. It is further supported by clinical evidence on the timing of diagnosis: a diagnosis of CAH after the first year of life—compared with diagnosis within the first month—was associated with a higher risk of TART development, whereas neonatal screening, by enabling earlier diagnosis, was associated with a lower risk of subsequent TART detection [9]. Taken together, these observations suggest that prenatal or early postnatal ACTH exposure may be critical for TART initiation, presumably through the establishment or expansion of ACTH-sensitive testicular cell populations, whereas cumulative ACTH exposure and other hormonal signals may contribute to subsequent tumor growth [2,7]. Sporadic reports have described TARTs in males with 11β-hydroxylase deficiency and 3β-hydroxysteroid dehydrogenase type 2 deficiency, in each case limited to single patients or very small case series, a pattern in keeping with sustained ACTH excess as a common pathogenetic mechanism shared across CAH subtypes [10,11].

### 3.2. Determinants of TARTs

The likelihood of TART development is modulated by several clinical and genetic factors, the most relevant of which are summarized below.

*CAH phenotype*/*genotype*. A prospective longitudinal study confirmed the SW phenotype as a significant risk factor for TART development, with a markedly increased prevalence compared with the simple virilizing form (68% vs. 32%) [12]. Genotype also emerged as a significant determinant of risk: CYP21A2 genotypes are conventionally classified into severity groups based on predicted residual 21-hydroxylase activity [13]. Null and group A genotypes are generally associated with the most severe salt-wasting phenotype and appear to carry the highest TART risk, whereas group B and group C genotypes are associated with progressively milder phenotypes and lower risk [12,14].

*Chronic poor hormonal control*. In this context, poor hormonal control refers to persistent or recurrent biochemical evidence of inadequate suppression of the HPA axis on serial testing, typically reflected by repeatedly elevated ACTH, 17-OHP, and androstenedione rather than isolated abnormal values which carry limited predictive value. Persistent biochemical evidence of poor hormonal control, including repeatedly elevated ACTH, 17-OHP, and androstenedione, has been associated with TART development in several cohorts. Single isolated hormone values should, however, be interpreted cautiously, since TART risk appears more closely related to chronic or cumulative ACTH exposure and overall longitudinal disease control. Within this framework, persistently elevated ACTH levels > 20 ng/dL has been reported to discriminate patients with TARTs from those without [15], supporting raised ACTH as a practical indicator of suboptimal disease control. Δ4-androstenedione has likewise proven informative, emerging as one of the most predictive parameters even when measured two years before the diagnostic ultrasound [14]; across several studies, androstenedione concentrations were significantly higher in patients with TARTs than in those without, frequently exceeding the upper reference limit and reflecting poor hormonal control at the time of evaluation [12,16,17,18]. Nevertheless, TARTs are also found in well-controlled patients, confirming a multifactorial pathogenesis [19].

*Puberty, LH and treatment adherence*. Most detected TARTs occur in pubertal patients [15,20], a peak in prevalence possibly related to the LH receptors expressed in TART tissue [15]. Puberty is also frequently marked by declining treatment adherence, which is rarely measured objectively in TART cohorts but is indirectly supported as a modifier of hormonal control and, in turn, of TART risk by its temporal overlap with peak TART detection and with recurrent biochemical breakthrough despite adequate prescribed doses [21]; because adherence is seldom formally assessed, its independent contribution remains difficult to quantify and warrants closer prospective evaluation. This peak may also partly reflect an ascertainment effect, as the increase in testicular volume eases ultrasonographic detection, consistent with recommendations to begin surveillance in adolescence [22].

## 4. Screening for TARTs: Evidence and Recommendations

### 4.1. Rationale for Earlier Surveillance of TARTs

The 2018 Endocrine Society guideline recommends periodic testicular ultrasound in males with classic CAH from adolescence onward, leaving the exact timing and interval to clinical judgement [22]. Accumulating pediatric evidence, however, indicates that TARTs are detectable well before puberty in a non-negligible proportion of boys with classic CAH—particularly those with severe phenotypes or suboptimal hormonal control [12,20,23]. Although prevalence rises with age and peaks in adolescence and adulthood, systematically screened cohorts have identified a substantial share of lesions in prepubertal boys, including cases in early childhood, implying that an adolescence-based strategy may miss early disease [16,24,25,26]. Several pediatric cohorts illustrate this prepubertal onset. Martinez-Aguayo et al. detected TARTs in roughly 21% of young boys with CAH, predominantly those with SW disease, in whom higher 17-OHP concentrations indicated poorer hormonal control [27]; a comparable overall prevalence (24%) has been reported by Mendes-Dos-Santos et al., including several prepubertal cases with a youngest affected age of 5 years, and TART development was again linked to poor disease control [21]. Prepubertal TARTs in 21-OHD have been documented with the youngest affected patient aged 6.2 years [28], and affected children as young as 4 years—most with suboptimal metabolic control—have likewise been described [20]. In a further cohort of Kim et al., TARTs were found in about 11% of young boys (youngest aged 6.6 years); all affected boys had classic CAH and advanced bone age, with positivity associated with a higher fludrocortisone dose [29]. Larger datasets reinforce this early-risk pattern. In the I-CAH registry, risk was higher with the salt-wasting phenotype, diagnosis delayed beyond the first year of life, and absence of neonatal screening [9]. In the longitudinal NIH cohort, nearly half of TART-positive patients (48%) were prepubertal at first detection; the estimated cumulative risk of TARTs before age 10 was 22%, with earlier onset in salt-wasting 21-OHD and null CYP21A2 genotypes [12]. The rationale for earlier surveillance is not merely earlier detection per se, but the possibility of identifying small, clinically silent, non-palpable lesions at an earlier stage, before chronic obstruction, fibrosis, and irreversible impairment of spermatogenesis have occurred, when tumor growth may still be modifiable through optimization of hormonal control [2,3,30,31]. This is particularly relevant in prepubertal boys, in whom semen analysis is not feasible and ultrasound represents the only practical tool to detect subclinical testicular involvement and stratify future fertility risk.

### 4.2. Diagnostic Modalities

Because TARTs are typically located within or near the rete testis, early progression is often clinically silent: small lesions are usually non-palpable and may remain asymptomatic for years. Ultrasound can detect lesions of only a few millimeters, whereas palpation generally identifies only larger masses [2,30]. Without imaging, diagnosis may therefore be delayed until lesions are larger, bilateral, or associated with advanced testicular involvement [32,33]. Scrotal ultrasound is the modality of choice for detecting and monitoring TARTs: it is fast, widely available, radiation-free, inexpensive, and able to identify nodules of only a few millimeters [34]. Its principal clinical strength lies in the longitudinal assessment of tumor evolution—regression, stability, or progression—and the typical sonographic features are summarized in Table 1.

A recent contribution to TART imaging diagnostics is the proposed four-level sonographic staging system (S-stage 0–3), validated against the corresponding histological staging system (H-stage 1–5) [12,19]. This staging system provides a practical clinical tool for monitoring disease burden and informing treatment decisions.

Stage 0: Normal testis [H-stage 1: TART cells confined to the rete testis but not detectable];

Stage 1: Single hypoechoic nodule near the mediastinum testis [H-stage 2: TART cell hyperplasia/hypertrophy];

Stage 2: Two or more separate hypoechoic nodules near the mediastinum testis [H-stage 2];

Stage 3: Conglomerate hypoechoic mass near the mediastinum testis [H-stages 3 and 4: rete testis compression, fibrosis, and lymphocytic infiltration].

**Contrast-enhanced ultrasound (CEUS)**, performed with a second-generation contrast agent, may improve lesion detection. It is generally regarded as advantageous over conventional ultrasound, as it provides real-time imaging of blood flow down to the microvascular level and may therefore aid in the detection of small lesions (≤1 cm) [14,33].

**Elastography** complements conventional ultrasound by quantifying tissue stiffness. TARTs have been reported to be stiffer than normal testicular parenchyma on elastography, with stiffness and vascularity indices decreasing after glucocorticoid therapy; findings that suggest a potential role for elastography in monitoring treatment response [37].

**MRI** appears comparable to ultrasound for TART detection and monitoring but adds no diagnostic information [34,38,39]. The typical pattern is hypointense on T2 (reflecting the fibrous component), iso- to hyperintense on T1, with homogeneous gadolinium enhancement more intense than that of the normal parenchyma and no capsule [34]. MRI may be indicated for: (1) pre-surgical planning (testis-sparing surgery); (2) lesions < 1 cm not adequately characterized by ultrasound or CEUS; (3) residual diagnostic uncertainty in patients not eligible for biopsy [14,32].

A comparative summary of the advantages and limitations of each modality is presented in Table 2.

*Differential diagnosis*. On ultrasound, TARTs must be distinguished from other testicular masses. Leydig-cell tumors are typically unilateral and well-circumscribed, in contrast to the usually bilateral, multilobulated pattern of TARTs; at the molecular level, TARTs express adrenal markers (CYP11B1, CYP21A2, and MC2R) and lack the Leydig-cell maturation marker INSL3 [40]. Testicular lymphoma or leukemic infiltration presents as diffuse hypoechoic lesions but is distinguished by its hematological context and rapid growth. Microlithiasis shows diffuse hyperechoic foci without a discrete mass, and orchitis is accompanied by pain, fever, and increased vascularity that resolve with treatment, unlike the indolent course of TARTs.

### 4.3. Risk-Stratified Screening Framework

Based on the determinants associated with the earlier appearance of TARTs, a risk-adapted approach to surveillance may be considered in which the timing and frequency of testicular ultrasound are individualized according to phenotype, genotype, and quality of hormonal control (Table 3). This framework should be interpreted as a pragmatic synthesis of the available observational evidence rather than as a formal screening recommendation and requires prospective validation.

Across all categories, screening frequency may reasonably be intensified during puberty and early adolescence—the period of peak prevalence—and may be relaxed in well-controlled adults without a prior history of TART.

Independent of age, the following features should heighten clinical suspicion and support consideration of more intensive surveillance: a SW phenotype; a severe genotype (null or group A); persistent biochemical evidence of poor hormonal control (repeatedly elevated ACTH, 17-OHP, and androstenedione); and poor adherence to glucocorticoid therapy [2,12,14,15,16].

*Rationale for the proposed intervals*. The risk stratification itself is well supported: multiple independent cohorts consistently link the salt-wasting phenotype and severe (null/group A) genotypes to earlier, more frequent TART detection [9,12,20,27,28,29]. The specific starting ages and intervals, however, are pragmatic extrapolations rather than validated thresholds: the 4–6-year starting age for high-risk patients reflects the youngest documented ages of TART detection in comparable cohorts (4–6.6 years) [20,28,29], while the 6–8-year threshold for intermediate risk allows a margin beyond this range, as prepubertal data for milder genotypes remain scarce. Surveillance intervals follow the same logic, tracking each category’s reported prevalence and pace of progression rather than any study that directly compared screening schedules.

## 5. Potential Clinical Implications of Earlier Screening and Final Considerations

Earlier screening for TARTs should not be viewed simply as diagnosing a benign testicular tumor at a younger age, but as a means of identifying an early marker of gonadal vulnerability in boys with classic CAH. In prepubertal patients, in whom semen analysis is not yet informative, ultrasound detection of small, non-palpable lesions may signal adrenal-rest activation and help to distinguish boys who require closer endocrine and reproductive follow-up from those who may safely undergo less intensive monitoring [12,19]. This is relevant because TART progression appears to be dynamic: early adrenal-rest hyperplasia may evolve into multilobular lesions within the rete testis, with subsequent tubular obstruction, subfertility or infertility, fibrosis and potentially irreversible testicular damage [2,19]. The value of earlier screening may therefore lie less in immediate intervention than in defining a longitudinal trajectory—establishing baseline testicular status before puberty, tracking lesion growth or stability, integrating imaging with disease-control and Sertoli-cell biomarkers, and identifying the optimal window for treatment optimization before structural damage becomes established [12,27]. Early detection may also help to curb indiscriminate glucocorticoid escalation, reserving treatment intensification for patients with demonstrable or progressive lesions or biochemical evidence of ACTH excess, and thereby limiting overtreatment, which may itself impair gonadotropin secretion and metabolic health [2,41]. Finally, recognizing TARTs before or around puberty allows timely fertility counselling, appropriately timed semen analysis, and early discussion of sperm cryopreservation in adolescents or young adults with persistent or progressive lesions [2,41].

The evidence on TARTs remains methodologically limited: most studies are retrospective and cross-sectional, with small, heterogeneous samples, and no randomized trials have addressed the optimal timing of ultrasound screening or the intensity of glucocorticoid treatment. Direct evidence that earlier screening improves long-term fertility is likewise scarce, and tumor regression following treatment intensification has been reported in only a subset of patients; the rationale for earlier screening is therefore prevention-oriented rather than grounded in definitive interventional outcomes [2,12,19,22]. Current recommendations consequently rest on observational data and expert consensus, and prospective multicenter studies with standardized protocols are needed. Implementing earlier, risk-based screening also faces practical constraints, including uneven access to experienced pediatric sonographers, unassessed added costs, potential parental anxiety, and the risk of overdiagnosing non-progressive lesions; these should be weighed alongside the framework’s potential benefits. Within these limits, the cumulative evidence portrays TARTs as a frequent, often clinically silent and potentially progressive complication of classic CAH, with relevant implications for fertility. This evidence supports a measured shift toward earlier, risk-based ultrasound assessment, specifically a baseline scrotal ultrasound before puberty in higher-risk boys: those with a salt-wasting phenotype, severe CYP21A2 genotypes, delayed diagnosis or persistent biochemical evidence of poor disease control. We regard this as a reasonable refinement of current guideline-based practice, with the optimal starting age and interval to be established through prospective, multicenter validation.

## Figures and Tables

**Table 1:** Typical ultrasound features of TARTs.

Parameter	Typical Features in TARTs
Echogenicity	Predominantly hypoechoic; may be iso-hypoechoic; hyperechogenicity with posterior acoustic shadowing in advanced, fibrotic tumors [2,35]
Location	Near the mediastinum testis (rete testis), in the middle third of the testis; epididymal involvement rarely reported [34,35,36]
Laterality	Frequently bilateral (≈81%) [2,35]
Number and shape	Multiple; homogeneous and well-delineated from the parenchyma; round/oval or lobulated; no capsule or pseudo-capsule [2,34,35,36]
Color Doppler	Variable vascularity (hypo-, iso-, or hypervascular); vessels show no deviation in their course without displacement or change in caliber, helping to differentiate TARTs from malignant tumors [34,35,36]
Calcifications	Described in advanced or long-standing lesions, including coarse calcifications and microlithiasis [34]
Testicular volume	Typically normal; not increased [27,36]
Palpability	Only when ≥2 cm (a minority, especially in children) [2]

TART, testicular adrenal rest tumor.

**Table 2:** Comparison of imaging modalities for the detection and monitoring of testicular adrenal rest tumors (TARTs).

Modality	Advantages	Limitations
Ultrasound (US)	First-line; fast, inexpensive, radiation-free; detects lesions of only a few millimeters; enables longitudinal monitoring	Operator-dependent; limited soft-tissue characterization
CEUS	Improves detection of small (≤1 cm) lesions via real-time microvascular flow imaging	Limited pediatric evidence; no dedicated diagnostic-accuracy studies in TARTs to date
Elastography	Non-invasive assessment of tissue stiffness; potential marker of treatment response	Reproducibility and normative pediatric values not established
MRI	Sensitivity reportedly comparable to US; superior soft-tissue contrast; useful for pre-surgical planning	Costly; limited pediatric availability, often requires sedation; reserved for selected cases

**Table 3:** Proposed pragmatic risk-stratified framework for testicular ultrasound surveillance of TARTs in boys with CAH. This framework represents the authors’ synthesis of the available observational evidence and should not be interpreted as a formal guideline; prospective validation is required.

Risk Category	Patient Profile	Suggested Surveillance Starting Age	Suggested Surveillance Interval
HIGH RISK	21-OHD SW; null/group A genotype (e.g., large deletions/conversions, I2splice, Q318X, R356W, E6 cluster, or similarly severe variants); poor hormonal control (persistently elevated ACTH, 17-OHP, androstenedione, and/or plasma renin activity)	Consider baseline ultrasound in early childhood, approximately 4–6 years, or at diagnosis if delayed	Annually through childhood; consider every 1–2 years after puberty if repeatedly clinically stable
INTERMEDIATE RISK	21-OHD SV; group B genotype (I172N); variable metabolic control	6–8 years	Every 1–2 years; closer to annual follow-up if biochemical control is unstable or clinical risk factors accumulate
LOW RISK	21-OHD NC; group C genotype; stable control	Routine screening is less supported; if pursued, from adolescence (earlier if control worsens)	Every 2–3 years; reassess if hormonal control changes

SW, salt-wasting; SV, simple virilizing; NC, non-classic; 21-OHD, 21-hydroxylase deficiency; ACTH, adrenocorticotropic hormone; 17-OHP, 17-hydroxyprogesterone; The most severe CYP21A2 genotypes correspond to the null and group A categories, whereas group B (e.g., I172N) and group C variants carry progressively lower risk.

## Data Availability

Data sharing is not applicable. No new data were created or analyzed in this study.

## Abbreviations

The following abbreviations are used in this manuscript:

CAH	Congenital adrenal hyperplasia
SW	Salt wasting
SV	Simple virilizing
17-OHP	17-hydroxyprogesterone
21OHD	21-hydroxylase deficiency
ACTH	Adrenocorticotropic hormone
CEUS	Contrast-enhanced ultrasound
CYP21A2	Steroid 21-hydroxylase gene
LH	Luteinizing hormone
MRI	Magnetic resonance imaging
NC	Non-classic
TART	Testicular adrenal rest tumor
US	Ultrasound
